# Hematopoietic Stem Cells and Mesenchymal Stromal Cells in Acute Radiation Syndrome

**DOI:** 10.1155/2020/8340756

**Published:** 2020-08-08

**Authors:** Liren Qian, Jian Cen

**Affiliations:** Department of Hematology, The Sixth Medical Center, Chinese PLA General Hospital, Fucheng Road #6, Beijing 100048, China

## Abstract

With the extensive utilization of radioactive materials for medical, industrial, agricultural, military, and research purposes, medical researchers are trying to identify new methods to treat acute radiation syndrome (ARS). Radiation may cause injury to different tissues and organs, but no single drug has been proven to be effective in all circumstances. Radioprotective agents are always effective if given before irradiation, but many nuclear accidents are unpredictable. Medical countermeasures that can be beneficial to different organ and tissue injuries caused by radiation are urgently needed. Cellular therapy, especially stem cell therapy, has been a promising approach in ARS. Hematopoietic stem cells (HSCs) and mesenchymal stromal cells (MSCs) are the two main kinds of stem cells which show good efficacy in ARS and have attracted great attention from researchers. There are also some limitations that need to be investigated in future studies. In recent years, there are also some novel methods of stem cells that could possibly be applied on ARS, like “drug” stem cell banks obtained from clinical grade human induced pluripotent stem cells (hiPSCs), MSC-derived products, and infusion of HSCs without preconditioning treatment, which make us confident in the future treatment of ARS. This review focuses on major scientific and clinical advances of hematopoietic stem cells and mesenchymal stromal cells on ARS.

## 1. Introduction

Nuclear technology has been widely used in different fields, like medicine, industry, agriculture, military, and medicine. Exposure to radiation or nuclear leakage is sometimes unavoidable and potentially catastrophic. More than 400 radiological accidents have happened since the middle of the 20th century [1], and thousands of persons have been injured by irradiation. It is reported that over 600 of the 10 million sealed radioactive materials used worldwide have been lost or stolen [2, 3]. It can be inferred that there may still be parts of the lost radioactive materials that are undocumented. With the increasing number of conflicts between countries and terrorist threats, and with the increasing application of radiotherapy in clinics, radiotherapy accidents like radiation overdose and nuclear leakage caused by machine malfunction are also not rare [4]; thus, there is an unprecedented urgency to develop new methods to treat acute radiation syndrome (ARS). In recent years, many new radioprotectants like antioxidants and toll-like receptor 5 agonist have been found to be effective against radiation [5–15]. Radioprotectants are supposed to exert their efficacy if present before irradiation. However, nuclear leakage accidents are always unpredictable. Accidents from medical nuclear devices, the collapse of nuclear power plants caused by natural calamities, and the explosion of nuclear weapons may cause a huge loss of life and a considerable number of injuries. Developing new therapeutic methods to treat the injuries caused by irradiation is quite essential. Stagnation in supportive therapy on ARS is the major current situation [16]. Through in vivo studies, it has been found that cytokines like granulocyte colony-stimulating factor (G-CSF), granulocyte-monocyte colony-stimulating factor (GM-CSF), pegylated G-CSF (pegfilgrastim), interleukin-11, interleukin-3, and erythropoietin can either reduce the duration of pancytopenia or improve outcomes [16–21]. Other supportive treatments include blood product transfusion, anti-infective therapy, and antiemetic drugs. These treatments are mostly symptomatic approaches. However, etiological treatments are usually not easy to implement, and studies of etiological treatments of ARS lag further behind. A manual entitled “Medical Management of Radiation Accidents: Manual on the Acute Radiation Syndrome” provide recommendations for the medical management of radiation accident victims [22] based on clinical archives and preclinical experiments. The role of hematopoietic stem cells (HSCs) has attracted researchers' attention since 1951 when Lorenz et al. found that infusion of bone marrow cells could prolong the survival time of irradiated mice [23, 24], while the role of mesenchymal stromal cells (MSCs) in ARS has just been found in recent decades in mice, for their power to migrate to the site of injury [25–27]. MSCs home in to injured tissues when coinfused with HSCs to treat a radiation-induced multiorgan failure syndrome [28]. The objective of this review was to offer an overview of the major scientific and clinical advances of HSCs and MSCs as therapeutic countermeasures against irradiation.

## 2. Acute Radiation Syndrome

ARS can be clinically manifested as a continuous progression, according to the radiation dose, from nausea and vomiting in the prodromal stage to a hematopoietic, gastrointestinal tract, cutaneous, or neurovascular syndrome [29, 30]. ARS has four different phases: the prodromal phase, the latent phase, the illness phase, and one phase of recovery or death. Time to death is very often dictated by type of organ injury. When an individual is exposed to a dose of 10-20 Gy or higher, prodromal symptoms will appear within 1 to 72 hours, including fever, loss of appetite, nausea and vomiting, electrolyte disturbances, and even hypotension, loss of consciousness, and finally death in a few days. A severe rapid prodromal stage suggests a higher absorbed dose and predicts poor clinical prognosis. Target organ damage occurs subsequently after the prodromal period. The severity of radiation injuries depends on the radiation dose incurred, the dose rate, the radiosensitivity of affected tissues and organs, and the area and extent to which the body has been exposed [31]. METREPOL clinically divides hematopoietic ARS into four grades (H1-H4) based on patients' peripheral blood cells over 60 days after irradiation [32]. H1 represents mild damage which need no specific therapy, and H2-H4 represents moderate, severe, and irreversible damage, respectively [32, 33]. The accident at the Chernobyl Nuclear Power Plant on 26 April 1986 resulted in the hospitalization of 237 patients identified as severely overexposed persons. ARS was diagnosed in 134 persons admitted to the specialized hospitals in Moscow and Kyiv. Among them, 28 died within three months of ARS associated with extensive local radiation burns combined with thermal burns. ARS was not confirmed in another 103 hospitalized patients [34].

**Figure 1:**
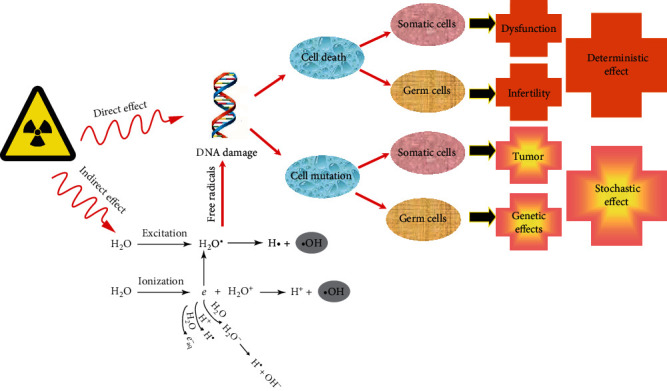
Biological effects of radiation.

The effects of ionizing radiation on biomolecules can be divided into direct and indirect effects (Figure 1) [35]. Direct effect means the energy of radiation rays may transfer to biomolecules directly causing ionization and excitation. Radiation rays can also act on water molecules, causing activation of water molecules and formation of free radicals. These activated products then act on other biomolecules. The effects produced in this way are called indirect effects. Because the body and cells contain a large amount of water, most of the radiant energy is absorbed by water resulting in decomposition of water molecules to generate a large number of free radicals [5]. They can damage various biological macromolecules in the body. This indirect effect causes damage to the body accounting for about 80% of the radiation damage [9, 36]. DNA can be damaged by irradiation through either direct or indirect action [37]. During the repair process of DNA post irradiation, some DNA injuries can completely recover by complex metabolic and immunological mechanisms, while some DNA injuries can recover but with mistakes in DNA repair like nonclonal genetic deletion and genetic insertion [38]. Accumulated gene mutation or instabilities may lead to malignant diseases several years later. Stem cell therapy has been proven effective in genetic diseases, like sickle cell disease, thalassemia, and immunodeficiency diseases [39–41]. Oxidative stress caused by free radicals generated from radiation plays a major role in radiation-induced injury. Besides, the reactive oxygen/nitrogen species that results from irradiation of normal tissues can be used as intracellular and intercellular signals to change cell and tissue functions. The increase of free radicals can lead to changes in molecular pathways. These signaling pathways play an important role in the pathogenesis of many pathological states, including inflammation, cancer, and diseases of some organs, and can promote the process of aging [42].

HSCs and MSCs are two types of cells much more successful in clinical applications that have also been proven to be effective in treating ARS either in preclinical models or in clinical case studies [30, 43].

## 3. Hematopoietic Stem Cell Transplantation in ARS

HSCs are multipotential stem cells with the ability to differentiate and self-renew. Because radiation may cause bone marrow failure, the question of whether infusion of bone marrow cells can be engrafted and have the ability to self-renew and differentiate to peripheral blood cells has aroused the thinking of early scientists.

As early as 1951, Lorenz et al. have found that infusion of bone marrow cells has a therapeutic effect on lethal doses of radiation [23]. They found that infusion of bone marrow cells from homologous animals 10 to 15 minutes after a lethal dose of radiation can reduce the mortality of mice to less than 30% and the mortality of guinea pigs to less than 50%. Infusion of bone marrow cells from heterologous animals also has a therapeutic effect, which can reduce the mortality to about 60% [24]. The cause of higher mortality with a heterologous transplant of bone marrow is probably caused by graft-versus-host disease (GVHD) after transplantation, when there was still no clear concept of GVHD. Since then, with the development of HSCT, the role of HSC in radiation became an interesting area for scientific researchers. In the following years, many preclinical studies have repeatedly confirmed the role of HSC in radiation and tried to explain its mechanism [44, 45]. Although HSCT has shown exciting therapeutic effects in preclinical animal experiments with acute radiation injury, its effects are still controversial in clinical applications. The earliest HSCT in clinical patients dates back to the middle of the last century. Bone marrow cells were transfused into 4 patients in one nuclear accident in 1958 [46]. In the Yugoslavian accident, 6 persons were exposed to radiation; 1 died, and 4 of the recovered victims received total allogeneic bone marrow injections. The victims presented not only hematopoietic syndrome but also gastrointestinal tract syndrome and neurovascular syndrome. Although the patients who have been infused with bone marrow cells have had a transient graft, the implantation has not significantly restored peripheral blood cells. The author thought it might be due to the late timing of infusion [46]. Temporary implantation may indicate that bone marrow cell infusion may have potential therapeutic effects on acute radiation injury. To date, about 50 patients with acute radiation sickness have been treated with allogeneic hematopoietic stem cell transplants [38]. However, the median survival time of these patients has not yet exceeded one month [1, 47]. In addition, patients who have had a longer or even more than one-year survival period have shown autologous hematopoietic recovery, which has led researchers to question the role of HCT in acute radiation injury.

Radiation can also cause severe damage to multiple systems and organs throughout the body, such as damage to the heart and nervous system. Radiation can cause damage to heart pump function and myocardium. The most serious type of radiation-induced heart disease (RIHD) seems to be a type of myocardial degeneration, i.e., perivascular and interstitial fibrosis 6-10 weeks after radiation [9]. For example, in patients with clinical chest tumors undergoing radiation therapy, radiation can affect the heart, blood vessels, lungs, and spinal cord, resulting in the remodeling of related tissue cells and adverse side effects. This complex process is mediated by the complex biological effects of radiation. Radiation can cause inflammation, endothelial cell dysfunction, and thrombosis and eventually lead to organ dysfunction and heart failure in the form of pathological entities of RIHD [42]. Radiation may also induce spinal cord damage which is relatively rare and usually called radiation myelopathy (RM) due to radiation-induced cell apoptosis, like oligodendrocytes and endothelial cells [48]. Interestingly, HSCs have also been reported to regenerate nonhematopoietic tissues in recent decades, like myocardium and nerves [49–51]. To date, 29 clinical trials can be found for stem cell transplantation and myocardial infarction, including 5 closed. Orlic et al. found that 68% of newly formed myocardial tissue formed in the infarcted myocardial area 9 days after transplantation of bone marrow cells from transgenic mice [52]. Following the study, some clinical trials have been initiated using stem cell transplantation to treat myocardial infarction [50, 51]. However, this opinion was opposed by Balsam et al. [49]. Their findings were inconsistent with Orlic et al. They used a fluorescent labeling method to track the differentiation of HSCs from transgenic mice in ischemic myocardium. They found that the cells that differentiated from the transplanted HSCs in the myocardium did not express the cardiomyocyte markers, but instead appeared as the hematopoietic marker CD45 and the myeloid marker Gr-1, a protein also known as Ly-6G/Ly-6C. They explained that the differences between their study and Orlic et al.'s study may be due to an “anaesthetic and/or surgical technique.” They also pointed out that Orlic et al. did not stain the transplanted cells for additional hematopoietic markers, like CD45 or Gr-1, which may lead to a different conclusion. They also called for caution in the use of HSCs in the treatment of myocardial infarction in clinical trials, otherwise it is easy to delay the best time for patients.

Besides cardiomyocytes, many studies have confirmed that HSCs can differentiate into nerve cells [53–56]. Sigurjonsson et al. found that 4 to 9 days after CD34+ HSCs were implanted into lesions of the developing spinal cord, some of the implanted cells began to differentiate into neural cells expressing NeuN and MAP2. While HSCs differentiate into neural tissue cells, their CD34+ expression gradually disappeared [57]. They also found that the spinal microenvironment and cell differentiation efficiency are closely related. Although there are still debates as to whether HSCs can be regenerated into nonhematopoietic cells, no matter from the basic experiments or clinical trials, this has made us look forward to this application. This cannot help, but let us consider whether the use of hematopoietic stem cell transplantation for ARS is not only for the reconstruction of the hematopoietic system and the immune system but also for the regeneration of other organ cells.

There are also some limitations for using HSCT to treat ARS. Because patients often cannot be expected to be irradiated before accepting a nuclear accident, few patients have stored their own HSCs, which makes the application of autologous stem cell transplantation in ARS almost impossible. Engraftment syndrome (ES) after allogeneic HSCT is increasingly diagnosed [58], occurring independently of GVHD in 79% of the patients [59], which is manifested as fever, pulmonary vascular leak, rash, and organ dysfunction. Allogeneic HSCT also has some intractable limitations: (1) lack of donor sources for HLA-matched sibling HSCs; (2) the preconditioning before hematopoietic stem cell transplantation will cause a secondary blow to the body and can cause serious infections, bleeding, organ failure, and other complications; (3) immunosuppressants used to prevent graft-versus-host diseases (GVHD) after allogeneic HSCT can cause serious infections and even threaten life; (4) GVHD can cause damage to multiple organs and tissues throughout the body, which can make a third blow to the body; and (5) radiation often causes damage to multiple organs throughout the body, not only the hematopoietic system and the immune system. The above factors combined with the current small number of patients with ARS, lack of clinical experiences, and other factors have led to the limitation of the application of HSCT in ARS, and to the current insufficient success rate. The above problems are also the next steps for radiation specialists and hematologists. I believe that if the above problems are solved well, HSCT will greatly improve the survival time and life quality of patients with ARS. The emergency treatment of populations requires the availability of ready-to-use frozen products to treat a group of individuals. “Drug” stem cell banks obtained from clinical grade human induced pluripotent stem cells (hiPSCs) will make it possible to produce stem cells of different types to treat the population [60]. HiPSCs from these “universal” donors are already available (http://www.gait.global/).

## 4. Mesenchymal Stromal Cells in ARS

The mesenchymal “stem” cell, which was first reported by Friedenstein et al. in 1968 [61], has the ability to self-renew and differentiate into three kinds of cells, including osteoblasts, chondrocytes, and adipocytes. It is a kind of spindle-shaped plastic adherent cell, which is isolated from bone marrow or other sources [62]. Heterogeneous procedures for isolating and cultivating mesenchymal “stem” cells among laboratories have prompted the International Society for Cellular Therapy (ISCT) to issue criteria for identifying unique populations of these cells. Consequently, the isolation of mesenchymal “stem” cells according to ISCT criteria has produced heterogeneous, nonclonal cultures of stromal cells containing stem cells with different multipotent properties, committed progenitors, and differentiated cells [63]. This group of cells separated by plastic adherence does not have the homogeneity of stem cells, and the true stemness of stem cells should be more complicated. The current recognized function of this group of cells does not meet the criteria for stem cells. Therefore, it was recommended to use the term *mesenchymal stromal cell* which should be more suitable for this group of heterogeneous cells instead of the term *mesenchymal stem cell* [62].

Although there are not many studies and reports on the application of MSCs in ARS, some of the biological functions recognized so far can support its efficacy in ARS. Firstly, MSCs can directly and indirectly secrete many cytokines, such as interleukin- (IL-) 6, IL-7, IL-8, IL-11, IL-12, IL-14, IL-15, macrophage colony-stimulating factor (M-CSF), stem cell factor (SCF), granulocyte colony-stimulating factor (G-CSF), and granulocyte-macrophage colony-stimulating factor (GM-CSF). These cytokines play a key role in promoting hematopoiesis, tissue repair, and maintaining homeostasis [64]. Secondly, after infusion of MSCs, they can migrate to the injury site under chemotactic factors while maintaining their original functions to play a mediating role [25, 65]. They can also differentiate into injured tissue cells or promote the repair of tissues at the injury site, like the heart, nervous system, skin, bone, fat, cartilage, muscle, and intestine [43, 65–67]. Thirdly, MSCs have immunomodulatory, anti-inflammatory properties [68]. MSCs have been proven to exert therapeutic effects in graft-versus-host disease after allogeneic HSCT due to its immune modulation properties [69]. MSCs' role in ARS has attracted the attention of researchers, and its efficacy has also been confirmed in different organs [43]. In 2013, MSC therapy of refractory irradiation-induced colitis was safe and effective on pain, diarrhea, hemorrhage, inflammation, and fistulization accompanied by modulation of the lymphocyte subsets toward an increase in T regulatory cells and a decrease in activated effector T cells. Mesenchymal stem cells represent a safe therapy for patients with refractory inflammatory bowel diseases [70]. And MSC treatment induces stimulation of endogenous host progenitor cells to improve the regenerative process and constitutes an initial approach to arguing in favor of the use of MSCs to limit/reduce colorectal damage induced by radiation [71]. Furthermore, allogeneic MSCs can be used in irradiated patients without rejection, making them quicker and easier to use to treat a group of people immediately after an accident if ready-to-use cell banks are available.

## 5. MSCs in Hematopoietic ARS

In 2005, Mourcin et al. demonstrated that in vitro experiments, the coculture of MSCs and irradiated CD34+ cells can significantly increase the expansion of CD34+ cells. The number of CD34+ cells in the MSC group is 4.9 times that of the non-MSC group. From this article, it can be inferred that the infusion of MSCs after receiving radiation irradiation may promote the expansion of patients' own HSCs and promote the recovery of their hematopoietic function [72]. MSCs can restore the bone marrow microenvironment in order to sustain hematopoiesis as demonstrated by Fouillard et al. in a phase 1 clinical trial [73]. In 2010, Hu et al. reported that infusion of MSCs 4 h after irradiation can significantly accelerate the recovery of peripheral blood cells. On the 26th day, the white blood cell counts of mice in the MSC group could recover to 90% compared to that before irradiation, but the white blood cell counts can only be restored to 80% in the non-MSC group, and MSCs could significantly reduce the apoptosis of bone marrow cells. They also found that the survival rate of the 5 × 10^7^/kg MSC group was significantly higher than that of the 2.5 × 10^7^/kg and 1.5 × 10^8^/kg groups [66]. Their research also demonstrated the therapeutic effects of MSCs on acute radiation injury on the hematopoietic system and confirmed that the number of cells returned to MSCs should have a certain upper limit value. If the upper limit value is exceeded, the effect will have more disadvantages than benefits. Cell stimulating factors have been routinely used in ARS. In 2013, Shim et al. reported a study that compared human umbilical cord blood-derived MSCs (hUCB-MSCs) and granulocyte colony-stimulating factor (G-CSF) in bone marrow ARS. They found that hUCB-MSC-treated mice had significantly better peripheral blood leukocyte recovery than the G-CSF-treated mice post irradiation [74]. This paper suggests to us that MSCs can be used as an ideal method for complementary or combined cytokine therapy on ARS. There are other sources of MSCs used for bone marrow ARS. A recent research applied Wharton's jelly MSCs (WJ-MSCs) from human umbilical cord to the treatment of ARS and found that WJ-MSCs can significantly enhance spleen and bone marrow cell capacity [75]. The volume of bone marrow cells in mice of the combined WJ-MSCs and antibiotic group was more than twice the volume of bone marrow cells of mice in the simple irradiation group at 60 days after infusion. A comprehensive treatment combination of radioprotective agents before radiation and MSCs after radiation was also proven to have a good therapeutic effect on acute radiation injury on the hematopoietic system [76]. In 2019, Mahmoud et al. demonstrated that delivering silymarin to rats 3 days before radiation and MSCs 24 hours after radiation can significantly reduce the radiation injury on the hematopoietic system [76].

## 6. MSCs in Gastrointestinal ARS

In addition to its good therapeutic effects on radiation-induced bone marrow failure, MSCs also have potential efficacy on gastrointestinal ARS. Several teams have demonstrated that bone marrow-derived adherent stromal cells (BMASCs) can alleviate acute gastrointestinal radiation syndrome [77–79]. Saha et al. found that compared with the control group, the ability of the crypt epithelial cells in the BMASC group to synthesize DNA can be increased by nearly 2 times, and the number of Lgr5-positive crypt base columnar cells can be increased to 10 times that of the control group at 3.5 days post irradiation [77]. WJ-MSCs can also significantly protect the intestines of irradiated mice. In a research in 2020, Bandekar et al. found that the length of mice jejunum villi of the WJ-MSC and antibiotic group was significantly longer than that of the radiation-only group [75]. In the group that received WJ-MSCs at 24 h after irradiation, the length of the jejunum recovered almost back to its normal length. With the delay in WJ-MSC infusion time, the therapeutic effects gradually decreased [75].

## 7. MSCs in Cutaneous ARS

Radiation can also cause severe acute damage to the skin, often manifested as erythema, edema, ulcers, necrosis, and so on. Severe burns can occur, and high exposition can lead to amputation [80]. In 2007, Francois et al. reported that infusion of MSCs 24 h after irradiation can significantly reduce skin phenotypic score and wound size from one to eight weeks post irradiation [81]. Horton et al. also reported that MSCs can significantly reduce skin lesions caused by radiation. They found that the level of interleukin-10 (IL-10) in the skin tissue could be significantly increased, and the level of interleukin 1*β* (IL-1*β*) was significantly reduced 14 days after infusion of MSCs. They demonstrated that MSCs play a therapeutic role through tumor necrosis factor receptor 2 (TNF-R2) mediating the production of IL-10 [82].

Besides the organs above, MSCs have also been shown to have therapeutic effects in radiation damage to other organs, such as the lungs, nervous system, and glands [83–90]. Although the role of MSCs in acute radiation injury is clear, the mechanism is still not very clear. MSCs may provide protection against some radiation-induced organ injuries, like liver injury by an antioxidative process, vasculature protection, hepatocyte differentiation, and trophic effects [91]. Francois et al. found that infusion of BM-MSCs can reduce the mir-27b level of NOD/SCID mice liver exposed to radiation and increase the level of SDF1*α*, which can also reduce oxidative stress post irradiation and increase the level of Nfr2 and SOD genes by the ROS-Nfr2 pathway. The pathway reduces the production of ROS, thereby reducing the damage to the liver caused by irradiation [91]. The “niche” or microenvironment where stem cells are located has been identified as a key element driving MSC differentiation, migration, and proliferation [92].

Research by Yang et al. found that MSCs aggregated in the lungs 4 hours after infusion of MSCs, while they were not distributed to other tissues or organs, including bone marrow, and mostly cleared up 24 hours after infusion. The authors speculated that MSCs may have a therapeutic effect on ARS, not directly but through soluble factors [93]. However, the lung was found to promote platelet production, and it was found to produce hematopoietic progenitor cells in recent years [94]. Opposite to these findings, the other team has demonstrated that MSCs migrate to irradiated tissues and stay there until 15 days [95]. Whether MSCs promote the differentiation of hematopoietic progenitor cells into peripheral blood cells through the produced cytokines or directly act on hematopoietic progenitor cells in the lungs still needs further investigation. Saha et al. suggested that BMASC can activate the Wnt *β*-catenin signaling pathway exerting a therapeutic effect on ARS [77]. And in the research by Bandekar et al., they found that the therapeutic effect of WJ-MSCs on ARS was significantly weakened by knocking out Nrf-2 and knocking down G-CSF and IL-6. From this article, it can also be speculated that MSCs may treat ARS through secreting cytokines and signal regulation [75]. During the last 2 decades, many preclinical animal studies have shown that MSCs mainly accelerate angiogenesis and reepithelialization through the secretory activity of extracellular vesicles (EVs), control inflammation and antiapoptosis, protect vessels, and promote tissue regeneration, thereby repairing radiation-induced injury [96, 97]. The emergency treatment of populations exposed to radiation requires that the treatment measures are ready, and it would be wise to suggest keeping MSC-derived extracellular vesicles available [97].

MSC-derived products also have shown their efficacy in tissue repair, which may be used in radiation induce injury. The MSC-derived extracellular matrix (ECM) has been shown to have the ability to promote cell proliferation while retaining stem cell properties. ECM produced by young cells can rejuvenate senescent cells by increasing their proliferation rate and differentiation potential [98]. Besides MSC-derived ECM, MSC-derived trophic factors (TFs) can also stimulate cell regeneration, promote tissue recovery, and protect cells from further injury [99].

Gene therapy of MSCs also showed promise in radiation-induced injury. In 2012, Drouet et al. used Amaxa technology to nucleofect adipocyte-derived multipotent MSCs with mock and Sonic hedgehog (Shh) pIRES2 plasmids [100]. When the monkeys were exposed to radiation for 48 hours, they were treated with manipulated MSCs, showing good tolerance. Shh-MSCs show good effects on reducing the duration of radiation-induced pancytopenia and increasing the slope of recovery of polymorphonuclear cells and platelets. Riccobono et al. also reported the therapeutic potential of transfected adipocyte-derived stem cells (ADSCs) to cure cutaneous ARS in a minipig model. ADSCs were transiently transfected by electroporation with a plasmid coding for Sonic hedgehog, which showed that injection of low-dose transfected cells can repair skin injury caused by radiation, avoiding necrosis and uncontrollable pain [101].

## 8. Conclusion

Although the therapeutic effects of HSCs and MSCs have been proven in both basic and clinical studies, there are still many unresolved problems as mentioned above. If these problems are solved well, it is believed that the survival time and quality of life of ARS patients will be further improved. With the development of cell-based therapies, it can make up for many of the drawbacks of cell replacement therapy, and the coordinated development of the two may benefit radiation-injured patients. For example, in recent years, HSC- and MSC-derived products have opened the door to develop new and innovative methods to reverse tissue damage caused by radiation as an alternative to cell transplants [99, 102]. Moreover, with the further understanding of human tissues and organs, it is believed that the role of HSCs and MSCs in ARS will be further optimized in the future. For example, studies have also found that the niches of the human hematopoietic system are not saturated [103–107], and that the allogeneic HSCs infused can be implanted well. Then, the question is whether we can increase the infused cell number to increase its efficacy especially when the human body is not treated in time after receiving radiation. In addition, a series of issues needs to be further discussed, such as how effective is the combined infusion of HSCs and MSCs in patients; what are the order, time, and dose of the two kinds of infused cells; and what can be done with graft rejection [108]. In conclusion, no matter how many are the unknown factors, HSCs and MSCs are two important treatments for ARS. With the advancement of research, the expectations they bring to the treatment of ARS patients are still worth looking forward to.
